# Free Radicals, Antioxidants in Disease and Health

**Published:** 2008-06

**Authors:** Lien Ai Pham-Huy, Hua He, Chuong Pham-Huy

**Affiliations:** 1 *Department of Pharmacy, Lucile Salter Packard Children’s Hospital, Stanford University Medical Center, Palo Alto, CA, USA;*; 2 *Department of Analytical Chemistry, China Pharmaceutical University, Nanjing, China;*; 3 *Laboratory of Toxicology, Faculty of Pharmacy, University of Paris 5, Paris, France*

**Keywords:** free radicals, antioxidants, beneficial effects, deleterious effects, oxidative stress, diseases, health

## Abstract

Free radicals and oxidants play a dual role as both toxic and beneficial compounds, since they can be either harmful or helpful to the body. They are produced either from normal cell metabolisms in situ or from external sources (pollution, cigarette smoke, radiation, medication). When an overload of free radicals cannot gradually be destroyed, their accumulation in the body generates a phenomenon called oxidative stress. This process plays a major part in the development of chronic and degenerative illness such as cancer, autoimmune disorders, aging, cataract, rheumatoid arthritis, cardiovascular and neurodegenerative diseases. The human body has several mechanisms to counteract oxidative stress by producing antioxidants, which are either naturally produced in situ, or externally supplied through foods and/or supplements. This mini-review deals with the taxonomy, the mechanisms of formation and catabolism of the free radicals, it examines their beneficial and deleterious effects on cellular activities, it highlights the potential role of the antioxidants in preventing and repairing damages caused by oxidative stress, and it discusses the antioxidant supplementation in health maintenance.

## INTRODUCTION

Oxygen is an element indispensable for life. When cells use oxygen to generate energy, free radicals are created as a consequence of ATP (adenosine triphosphate) production by the mitochondria. These by-products are generally reactive oxygen species (ROS) as well as reactive nitrogen species (RNS) that result from the cellular redox process. These species play a dual role as both toxic and beneficial compounds. The delicate balance between their two antagonistic effects is clearly an important aspect of life. At low or moderate levels, ROS and RNS exert beneficial effects on cellular responses and immune function. At high concentrations, they generate oxidative stress, a deleterious process that can damage all cell structures (1-10). Oxidative stress plays a major part in the development of chronic and degenerative ailments such as cancer, arthritis, aging, autoimmune disorders, cardiovascular and neurodegenerative diseases. The human body has several mechanisms to counteract oxidative stress by producing antioxidants, which are either naturally produced in situ, or externally supplied through foods and/or supplements. Endogenous and exogenous antioxidants act as “free radical scavengers” by preventing and repairing damages caused by ROS and RNS, and therefore can enhance the immune defense and lower the risk of cancer and degenerative diseases (11-15).

The theory of oxygen-free radicals has been known about fifty years ago (4). However, only within the last two decades, has there been an explosive discovery of their roles in the development of diseases, and also of the health protective effects of antioxidants.

This mini-review deals with the taxonomy, the mechanisms of formation and catabolism of the free radicals, it examines their beneficial and deleterious effects on cellular activities, it highlights the potential role of the antioxidants in preventing and repairing damages caused by oxidative stress, and it discusses the advantages and inconveniences of the antioxidant supplementation in health maintenance.

## CHARACTERISTICS OF FREE RADICALS AND OXIDANTS

ROS and RNS are the terms collectively describing free radicals and other non-radical reactive derivatives also called oxidants. Radicals are less stable than non-radical species, although their reactivity is generally stronger.

A molecule with one or more unpaired electron in its outer shell is called a free radical (1-5). Free radicals are formed from molecules via the breakage of a chemical bond such that each fragment keeps one electron, by cleavage of a radical to give another radical and, also via redox reactions (1, 2). Free radicals include hydroxyl (OH^•^), superoxide (O_2_^•–^), nitric oxide (NO^•^), nitrogen dioxide (NO_2_^•^), peroxyl (ROO^•^) and lipid peroxyl (LOO^•^). Also, hydrogen peroxide (H_2_O_2_), ozone (O_3_), singlet oxygen (^1^O_2_), hypochlorous acid (HOCl), nitrous acid (HNO_2_), peroxynitrite (ONOO^–^), dinitrogen trioxide (N_2_O_3_), lipid peroxide (LOOH), are not free radicals and generally called oxidants, but can easily lead to free radical reactions in living organisms (8). Biological free radicals are thus highly unstable molecules that have electrons available to react with various organic substrates such as lipids, proteins, DNA.

## GENERATION OF FREE RADICALS AND OXIDANTS

Formation of ROS and RNS can occur in the cells by two ways: enzymatic and non-enzymatic reactions. Enzymatic reactions generating free radicals include those involved in the respiratory chain, the phagocytosis, the prostaglandin synthesis and the cytochrome P450 system (1-9). For example, the superoxide anion radical (O_2_^•–^) is generated via several cellular oxidase systems such as NADPH oxidase, xanthine oxidase, peroxidases. Once formed, it participates in several reactions yielding various ROS and RNS such as hydrogen peroxide, hydroxyl radical (OH^•^), peroxynitrite (ONOO^–^), hypochlorous acid (HOCl), etc. H_2_O_2_ (a non radical) is produced by the action of several oxidase enzymes, including aminoacid oxidase and xanthine oxidase. The last one catalyses the oxidation of hypoxanthine to xanthine, and of xanthine to uric acid. Hydroxyl radical (OH^•^), the most reactive free radical *in vivo*, is formed by the reaction of O_2_^•–^ with H_2_O_2_ in the presence of Fe^2+^ or Cu^+^ (catalyst). This reaction is known as the Fenton reaction (3-8). Hypochlorous acid (HOCl) is produced by the neutrophil-derived enzyme, myeloperoxidase, which oxidizes chloride ions in the presence of H_2_O_2_. Nitric oxide radical (NO^•^) is formed in biological tissues from the oxidation of L-arginine to citrulline by nitric oxide synthase (3-8).

Free radicals can be produced from non-enzymatic reactions of oxygen with organic compounds as well as those initiated by ionizing radiations. The nonenzymatic process can also occur during oxidative phosphorylation (i.e. aerobic respiration) in the mitochondria (4, 5, 8).

ROS and RNS are generated from either endogenous or exogenous sources. Endogenous free radicals are generated from immune cell activation, inflammation, mental stress, excessive exercise, ischemia, infection, cancer, aging. Exogenous ROS/RNS result from air and water pollution, cigarette smoke, alcohol, heavy or transition metals (Cd, Hg, Pb, Fe, As), certain drugs (cyclosporine, tacrolimus, gentamycin, bleomycin), industrial solvents, cooking (smoked meat, used oil, fat), radiation. (4-14). After penetration into the body by different routes, these exogenous compounds are decomposed or metabolized into free radicals.

## BENEFICIAL ACTIVITIES OF FREE RADICALS AND OXIDANTS

At low or moderate concentrations, ROS and RNS are necessary for the maturation process of cellular structures and can act as weapons for the host defense system. Indeed, phagocytes (neutrophils, macrophages, monocytes) release free radicals to destroy invading pathogenic microbes as part of the body’s defense mechanism against disease (5, 10). The importance of ROS production by the immune system is clearly exemplified by patients with granulomatous disease. These patients have defective membrane-bound NADPH oxidase system which makes them unable to produce the superoxide anion radical (O_2_^•–^), thereby resulting in multiple and persistent infection (4, 5). Other beneficial effects of ROS and RNS involve their physiological roles in the function of a number of cellular signaling systems (7-9). Their production by nonphagocytic NADPH oxidase isoforms plays a key role in the regulation of intracellular signaling cascades in various types of nonphagocytic cells including fibroblasts, endothelial cells, vascular smooth muscle cells, cardiac myocytes, and thyroid tissue. For example, nitric oxide (NO) is an intercellular messenger for modulating blood flow, thrombosis, and neural activity (7). NO is also important for nonspecific host defense, and for killing intracellular pathogens and tumors. Another beneficial activity of free radicals is the induction of a mitogenic response (7, 8). In brief, ROS/RNS at low or moderate levels are vital to human health.

## DELETERIOUS ACTIVITIES OF FREE RADICALS AND OXIDANTS AND PATHOGENESIS

When produced in excess, free radicals and oxidants generate a phenomenon called oxidative stress, a deleterious process that can seriously alter the cell membranes and other structures such as proteins, lipids, lipoproteins, and deoxyribonucleic acid (DNA) (5-10). Oxidative stress can arise when cells cannot adequately destroy the excess of free radicals formed. In other words, oxidative stress results from an imbalance between formation and neutralization of ROS/RNS. For example, hydroxyl radical and peroxynitrite in excess can damage cell membranes and lipoproteins by a process called lipid peroxidation. This reaction leads to the formation of malondialdehyde (MDA) and conjugated diene compounds, which are cytotoxic and mutagenic. Lipid peroxidation occurs by a radical chain reaction, i.e. once started, it spreads rapidly and affects a great number of lipid molecules (14). Proteins may also be damaged by ROS/RNS, leading to structural changes and loss of enzyme activity (9, 14). Oxidative damage to DNA leads to the formation of different oxidative DNA lesions which can cause mutations. The body has several mechanisms to counteract these attacks by using DNA repair enzymes and/or antioxidants (6-9). If not regulated properly, oxidative stress can induce a variety of chronic and degenerative diseases as well as the aging process and some acute pathologies (trauma, stroke).

### Cancer and oxidative stress

The development of cancer in humans is a complex process including cellular and molecular changes mediated by diverse endogenous and exogenous stimuli. It is well established that oxidative DNA damage is responsible for cancer development. (3, 4, 11). Cancer initiation and promotion are associated with chromosomal defects and oncogene activation induced by free radicals. A common form of damage is the formation of hydroxyled bases of DNA, which are considered an important event in chemical carcinogenesis (3, 9). This adduct formation interferes with normal cell growth by causing genetic mutations and altering normal gene transcription. Oxidative DNA damage also produces a multiplicity of modifications in the DNA structure including base and sugar lesions, strand breaks, DNA-protein cross-links and base-free sites. For example, tobacco smoking and chronic inflammation resulting from noninfectious diseases like asbestos are sources of oxidative DNA damage that can contribute to the development of lung cancer and other tumors (3, 6). The highly significant correlation between consumption of fats and death rates from leukemia and breast, ovary, rectum cancers among elderly people may be a reflection of greater lipid peroxidation (5, 10).

### Cardiovascular disease and oxidative stress

Cardiovascular disease (CVD) is of multifactorial etiology associated with a variety of risk factors for its development including hypercholesterolaemia, hypertension, smoking, diabetes, poor diet, stress and physical inactivity amongst others (2, 15, 16). Recently, research data has raised a passionate debate as to whether oxidative stress is a primary or secondary cause of many cardiovascular diseases (16). Further *in vivo* and *ex vivo* studies have provided precious evidence supporting the role of oxidative stress in a number of CVDs such as atherosclerosis, ischemia, hypertension, cardiomyopathy, cardiac hypertrophy and congestive heart failure (2, 5, 15, 16).

### Neurological disease and oxidative stress

Oxidative stress has been investigated in neurological diseases including Alzheimer’s disease, Parkinson’s disease, multiple sclerosis, amyotrophic lateral sclerosis (ALS), memory loss, depression (17-20). In a disease such as Alzheimer’s, numerous experimental and clinical studies have demonstrated that oxidative damage plays a key role in the loss of neurons and the progression to dementia (19). The production of ß-amyloid, a toxic peptide often found present in Alzheimer’s patients’ brain, is due to oxidative stress and plays an important role in the neurodegenerative processes (20).

### Pulmonary disease and oxidative stress

There is now substantial evidence that inflammatory lung diseases such as asthma and chronic obstructive pulmonary disease (COPD) are characterized by systemic and local chronic inflammation and oxidative stress (21-24). Oxidants may play a role in enhancing inflammation through the activation of different kinases and redox transcription factors such as NF-kappa B and AP-1 (23, 24).

### Rheumatoid arthritis and oxidative stress

Rheumatoid arthritis is an autoimmune disease characterized by chronic inflammation of the joints and tissue around the joints with infiltration of macrophages and activated T cells (4, 25, 26). The pathogenesis of this disease is due to the generation of ROS and RNS at the site of inflammation. Oxidative damage and inflammation in various rheumatic diseases were proved by increased levels of isoprostanes and prostaglandins in serum and synovial fluid compared to controls (26).

### Nephropathy and oxidative stress

Oxidative stress plays a role in a variety of renal diseases such as glomerulonephritis and tubulointerstitial nephritis, chronic renal failure, proteinuria, uremia (5, 27). The nephrotoxicity of certain drugs such as cyclosporine, tacrolimus (FK506), gentamycin, bleomycin, vinblastine, is mainly due to oxidative stress via lipid peroxidation (27-30). Heavy metals (Cd, Hg, Pb, As) and transition metals (Fe, Cu, Co, Cr)-induced different forms of nephropathy and carcinogenicity are strong free radical inducers in the body (11, 12).

### Ocular disease and oxidative stress

Oxidative stress is implicated in age-related macular degeneration and cataracts by altering various cell types in the eye either photochemically or nonphotochemically (31). Under the action of free radicals, the crystalline proteins in the lens can cross-link and aggregate, leading to the formation of cataracts (32). In the retina, long-term exposure to radiation can inhibit mitosis in the retinal pigment epithelium and choroids, damage the photoreceptor outer segments, and has been associated with lipid peroxidation (33).

### Fetus and oxidative stress

Oxidative stress is involved in many mechanisms in the development of fetal growth restriction and pre-eclampsia in prenatal medicine (34-37). Some reports indicate that blood levels of lipid peroxidation products (F2-isoprostanes, MDA) are elevated in pre-eclamptic pregnancy and intra-uterine growth retardation and it has been suggested that ROS/RNS play a role in the etiology of these diseases (35-37). In pregnancies complicated by pre-eclampsia, increased expression of NADPH oxidase 1 and 5 isoforms which are the major enzymatic sources of superoxide in the placenta is seen (37).

**Figure 1:**
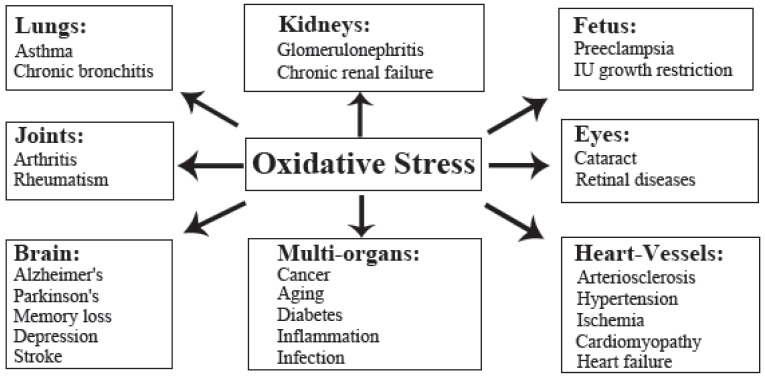
Oxidative stress-induced diseases in humans.

Finally, Figure 1 summarizes oxidative stress-induced diseases in humans.

## ANTIOXIDANTS AND HEALTH MAINTENANCE

The body has several mechanisms to counteract oxidative stress by producing antioxidants, either naturally generated in situ (endogenous antioxidants), or externally supplied through foods (exogenous antioxidants). The roles of antioxidants are to neutralize the excess of free radicals, to protect the cells against their toxic effects and to contribute to disease prevention.

### Antioxidant classification

Endogenous compounds in cells can be classified as enzymatic antioxidants and non-enzymatic antioxidants.

The major antioxidant enzymes directly involved in the neutralization of ROS and RNS are: superoxide dismutase (SOD), catalase (CAT), glutathione peroxidase (GPx) and glutathione reductase (GRx) (6-12). SOD, the first line of defense against free radicals, catalyzes the dismutation of superoxide anion radical (O_2_^•–^) into hydrogen peroxide (H_2_O_2_) by reduction. The oxidant formed (H_2_O_2_) is transformed into water and oxygen (O_2_) by catalase (CAT) or glutathione peroxidase (GPx). The selenoprotein GPx enzyme removes H_2_O_2_ by using it to oxidize reduced glutathione (GSH) into oxidized glutathione (GSSG). Glutathione reductase, a flavoprotein enzyme, regenerates GSH from GSSG, with NADPH as a source of reducing power. Besides hydrogen peroxide, GPx also reduces lipid or nonlipid hydroperoxides while oxidizing glutathione (GSH) (2, 5-10).

The non-enzymatic antioxidants are also divided into metabolic antioxidants and nutrient antioxidants. Metabolic antioxidants belonging to endogenous antioxidants, are produced by metabolism in the body, such as lipoid acid, glutathione, L-ariginine, coenzyme Q10, melatonin, uric acid, bilirubin, metal-chelating proteins, transferrin, etc (5, 6). While nutrient antioxidants belonging to exogenous antioxidants, are compounds which cannot be produced in the body and must be provided through foods or supplements, such as vitamin E, vitamin C, carotenoids, trace metals (selenium, manganese, zinc), flavonoids, omega-3 and omega-6 fatty acids, etc.

### Antioxidant Process

When an antioxidant destroys a free radical, this antioxidant itself becomes oxidized. Therefore, the antioxidant resources must be constantly restored in the body. Thus, while in one particular system an antioxidant is effective against free radicals, in other systems the same antioxidant could become ineffective. Also, in certain circumstances, an antioxidant may even act as a pro-oxidant e.g. it can generate toxic ROS/RNS (10). The antioxidant process can function in one of two ways: chain-breaking or prevention. For the chain-breaking, when a radical releases or steals an electron, a second radical is formed. The last one exerts the same action on another molecule and continues until either the free radical formed is stabilized by a chain-breaking antioxidant (vitamin C, E, carotenoids, etc), or it simply disintegrates into an inoffensive product. The classic example of such a chain reaction is lipid peroxidation. For the preventive way, an antioxidant enzyme like superoxide dismutase, catalase and glutathione peroxidase can prevent oxidation by reducing the rate of chain initiation, e.g., either by scavenging initiating free radicals or by stabilizing transition metal radicals such as copper and iron (10).

### Nutrient antioxidants

Antioxidants from our diet play an important role in helping endogenous antioxidants for the neutralization of oxidative stress. The nutrient antioxidant deficiency is one of the causes of numerous chronic and degenerative pathologies. Each nutrient is unique in terms of its structure and antioxidant function (6, 38).

**Vitamin E.** Vitamin E is a fat-soluble vitamin with high antioxidant potency. Vitamin E is a chiral compound with eight stereoisomers: α, β, γ, δ tocopherol and α, β, γ, δ tocotrienol. Only α-tocopherol is the most bioactive form in humans. Studies in both animals and humans indicate that natural dextrorotary d-α-tocopherol is nearly twice as effective as synthetic racemic dl-α-tocopherol (39). Because it is fat-soluble, α-tocopherol safeguards cell membranes from damage by free radicals. Its antioxidant function mainly resides in the protection against lipid peroxidation. Vitamin E has been proposed for the prevention against colon, prostate and breast cancers, some cardiovascular diseases, ischemia, cataract, arthritis and certain neurological disorders. (40). However, a recent trial revealed that daily α-tocopherol doses of 400 IU or more can increase the risk of death and should be avoided. In contrast, there is no increased risk of death with a dose of 200 IU per day or less, and there may even be some benefit (41). Although controversial, the use of long-term vitamin E supplementation in high dose should be approached cautiously until further evidence for its safety is available. The dietary sources of vitamin E are vegetable oils, wheat germ oil, whole grains, nuts, cereals, fruits, eggs, poultry, meat (6, 40). Cooking and storage may destroy natural d-α-tocopherol in foods (40).

**Vitamin C.** Vitamin C also known as ascorbic acid, is a water-soluble vitamin. It is essential for collagen, carnitine and neurotransmitters biosynthesis (42). Health benefits of vitamin C are antioxidant, anti-atherogenic, anti-carcinogenic, immunomodulator. The positive effect of vitamin C resides in reducing the incidence of stomach cancer, and in preventing lung and colorectal cancer. Vitamin C works synergistically with vitamin E to quench free radicals and also regenerates the reduced form of vitamin E. However, the intake of high doses of vitamin C (2000mg or more/day) has been the subject of debate for its eventual pro-oxidant or carcinogen property (42-43). Natural sources of vitamin C are acid fruits, green vegetables, tomatoes. Ascorbic acid is a labile molecule, therefore it may be lost from during cooking (43).

**Beta-carotene,** Beta-carotene is a fat soluble member of the carotenoids which are considered provitamins because they can be converted to active vitamin A. Beta-carotene is converted to retinol, which is essential for vision. It is a strong antioxidant and is the best quencher of singlet oxygen. However, beta-carotene supplement in doses of 20mg daily for 5-8 years has been associated with an increased risk of lung and prostate cancer and increased total mortality in cigarette smokers (44). Beta-carotene 20-30mg daily in smokers may also increase cardiovascular mortality by 12% to 26% (44). These adverse effects do not appear to occur in people who eat foods high in beta-carotene content. Beta-carotene is present in many fruits, grains, oil and vegetables (carrots, green plants, squash, spinach) (6).

**Lycopene.** Lycopene, a carotenoid, possesses antioxidant and antiproliferative properties in animal and *in vitro* studies on breast, prostate and lung cell lines, although anticancer activity in humans remains controversial (6, 45, 46). Lycopene has been found to be very protective, particularly for prostate cancer (46). Several prospective cohort studies have found associations between high intake of lycopene and reduced incidence of prostate cancer, though not all studies have produced consistent results (45). The major dietary source of lycopene is tomatoes, with the lycopene in cooked tomatoes, tomato juice and tomato sauce included, being more bioavailable than that in raw tomatoes (38).

**Selenium (Se).** Se is a trace mineral found in soil, water, vegetables (garlic, onion, grains, nuts, soybean), sea food, meat, liver, yeast (6). It forms the active site of several antioxidant enzymes including glutathione peroxidase. At low dose, health benefits of Se are antioxidant, anti-carcinogenic and immunomodulator (47). Selenium is also necessary for the thyroid function (48). Exceeding the Tolerable Upper Intake Level of 400 μg Se/day can lead to selenosis which is a selenium poisoning characterized by gastrointestinal disorders, hair and nail loss, cirrhosis, pulmonary edema and death (48). Selenium deficiency can occur in patients on total parenteral nutrition (TPN) and in patients with gastrointestinal disorders. In certain China areas with Se poor soil, people have developed a fatal cardiomyopathy called Keshan disease which was cured with Se supplement (48). The role of Se in cancer prevention has been the subject of recent study and debate. Results from clinical and cohort studies about cancer prevention, especially lung, colorectal, and prostate cancers are mixed (10, 48).

**Flavonoids.** Flavonoids are polyphenolic compounds which are present in most plants. According to chemical structure, over 4000 flavonoids have been identified and classified into flavanols, flavanones, flavones, isoflavones, catechins, anthocyanins, proanthocyanidins. Beneficial effects of flavonoids on human health mainly reside in their potent antioxidant activity (49). They have been reported to prevent or delay a number of chronic and degenerative ailments such as cancer, cardiovascular diseases, arthritis, aging, cataract, memory loss, stroke, Alzheimer’s disease, inflammation, infection. Every plant contains a unique combination of flavonoids, which is why different herbs, all rich in these substances, have very different effects on the body (50). The main natural sources of flavonoids include green tea, grapes (red wine), apple, cocoa (chocolate), ginkgo biloba, soybean, curcuma, berries, onion, broccoli, etc.

For example, green tea is a rich source of flavonoids, especially flavonols (catechins) and quercetin. Catechin levels are 4-6 times greater in green tea than in black tea. Many health benefits of green tea reside in its antioxidant, anticarcinogenic, antihypercholesterolemic, antibacterial (dental caries), anti-inflammatory activities (51).

**Omega-3 and omega-6 fatty acids.** They are essential long-chain polyunsaturated fatty acids because the human body cannot synthesize them. Therefore, they are only derived from food. Omega-3 fatty acids can be found in fat fish (salmon, tuna, halibut, sardines, pollock), krill, algae, walnut, nut oils and flaxseed. However, certain big fishes like tilefish, shark, swordfish are to be avoided because of their high mercury levels (52). There are three major dietary types of omega-3 fatty acids: eicosapentaenoic acid (EPA), docosahexaenoic acid (DHA) and alpha-linolenic acid (ALA). EPA and DHA are abundant in fish and are directly used by the body; while ALA is found in nuts and has to be converted to DHA and EPA by the body. Dietary sources of omega-6 fatty acids (linoleic acid) include vegetable oils, nuts, cereals, eggs, poultry. It is important to maintain an appropriate balance of omega-3s and omega-6s in the diet, as these two substances work together to promote health (52, 53). Omega-3 fatty acids help reduce inflammation, and most omega-6 fatty acids tend to promote inflammation. An inappropriate balance of these essential fatty acids contributes to the development of disease while a proper balance helps maintain and even improve health. A healthy diet should consist of about 2-4 times more omega-6s than omega-3s. In American diet, omega-6s are 14-25 times more abundant than omega-3s, that explains the rising rate of inflammatory disorders in the USA (52). Omega-3s reduce inflammation and prevent chronic ailments such as heart disease, stroke, memory loss, depression, arthritis, cataract, cancer. Omega-6s improve diabetic neuropathy, eczema, psoriasis, osteoporosis, and aid in cancer treatment (38, 52, 53).

Finally, some endogenous antioxidants such as L-arginine, coenzyme Q-10, melatonin are recently used as supplements for the prevention or treatment of some chronic and degenerative diseases (54-56). It is notified that the list of antioxidants cited here is not exhaustive.

### Antioxidant supplementation. Advantages and Inconveniences

Antioxidant supplements are compounds obtained either by extraction from natural foods or by chemical synthesis. Of course, they do not have the same composition as natural antioxidants in foods. Therefore, opinions are divided over whether or not antioxidant supplements offer the same health benefits as antioxidants in foods (6, 57-59). Even if antioxidant supplementation is receiving enthusiastic debate and is increasingly adopted in many industrial countries, supporting evidence is still ambiguous (5-59). Although many epidemiological data suggest that antioxidants may have a beneficial effect on many chronic diseases, the systematic use of supplements is hindered by several factors: the lack of prospective and controlled studies, the long-term effects and the dosages necessary for each type of diseases. Also, antioxidant supplements can act as pro-oxidants e.g. as oxidative stress inducers if they are consumed at levels significantly above the recommended dietary intakes (RDI). Like conventional medicines, dietary supplements may cause side effects, or interaction with another medication or supplement, that may make the health worse. However, dietary supplements can become necessary and useful in some particular situations, such as soldiers in front, sailors in ships, patients with gastrointestinal disorders, or people with low incomes, e.g. people who cannot afford a variety of vegetables, fruits, and/or sea foods. In these cases, taking one or two multivitamin with mineral tablets and fish oil capsules in RDI concentrations may be helpful to maintain good health. Taking supplements in high doses can be harmful and always consult a healthcare professional about combining a dietary supplement with a conventional medical treatment. If possible, it is best to get the antioxidants from a diet rich in fruits and vegetables rather than from supplements.

## CONCLUSION

The implication of oxidative stress in the etiology of several chronic and degenerative diseases suggests that antioxidant therapy represents a promising avenue for treatment. In the future, a therapeutic strategy to increase the antioxidant capacity of cells may be used to fortify the long term effective treatment. However, many questions about antioxidant supplements in disease prevention remain unsolved. Further research is needed before this supplementation could be officially recommended as an adjuvant therapy. In the meantime, it is reminded that avoiding oxidant sources (cigarette, alcohol, bad food, stress, etc) must be considered as important as taking diet rich in antioxidants. Indeed, our health also depends on our lifestyle choice.
